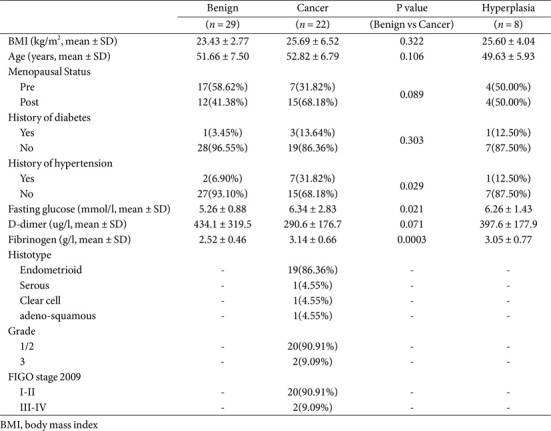# Distinct Microbial Signatures along the Female Reproductive Tract in Endometrial Cancer Patients

**DOI:** 10.4014/jmb.2503.03048

**Published:** 2025-08-26

**Authors:** Weicong Xu, Ayana Bayijuma, Jie Shi, Yujie Huang, Jingyu Wang, Kaiyou Fu, Baohong Wang, Yunxiao Zhou

**Affiliations:** 1 Department of Gynecology, The First Affiliated Hospital of Zhejiang University Medical College, Hangzhou 310003, P.R. China; 2 State Key Laboratory for Diagnosis and Treatment of Infectious Diseases, National Clinical Research Center for Infectious Diseases, Collaborative Innovation Center for Diagnosis and Treatment of Infectious Diseases, The First Affiliated Hospital, Zhejiang University School of Medicine, Hangzhou 310003, P.R. China; 3 Jinan Microecological Biomedicine Shandong Laboratory, Jinan 250117, P.R. China; 4 Shandong First Medical University & Shandong Academy of Medical Sciences, Jinan 250117, P.R. China

**Keywords:** Endometrial cancer, microbiome, female genital tract, 16S rRNA

## Abstract

Emerging evidence suggests that microbiota dysbiosis plays a critical role in the pathogenesis of endometrial cancer (EC), a leading cause of cancer-related deaths in women globally. However, few studies have simultaneously examined both the upper and lower genital tract microbiota in such individuals. In this study, we investigated alterations in microbiota composition across different parts of the female genital tract in a Chinese cohort of EC patients. Samples from 59 individuals (22 endometrial cancer patients; 8 endometrial hyperplasia patients, and 29 benign controls) were collected. In addition, a total of 58 vaginal swabs, 39 fallopian swabs, 16 peritoneal fluid samples, 36 urine swabs, and 34 endometrium samples were finally recruited. The composition of bacterial communities was determined by 16S ribosomal RNA Miseq sequencing. Specific taxa were significantly enriched in the EC group, including *Akkermansia muciniphila*, *Acinetobacter*, and *Pseudomonas* in the vagina, and *Pseudomonas*, *Bacillus*, *Streptomyces*, and *Burkholderia-Caballeronia-Paraburkholderia* in the endometrium. Meanwhile, *Acinetobacter* was positively correlated with fasting plasma glucose, while *Pseudomonas* was correlated with estrogen and progesterone receptor expression. An effective random forest model enabled us to distinguish EC patients from benign controls. Moreover, specific alterations in the composition and diversity of the reproductive tract microbiota in endometrial cancer patients were identified. Our findings suggest a potential link between microbiome alterations and estrogen and glucose metabolism in EC. However, further investigation is needed to elucidate the molecular mechanisms underlying these associations.

## Introduction

Endometrial cancer is one of the most prevalent cancers of the female reproductive system and shows an increasing tendency [1], posing a serious threat to women's lives and health. Risk factors for endometrial cancer confirmed by studies include exogenous or endogenous estrogen due to obesity [2], early menarche and late menopause [3], advanced age [4], diabetes mellitus [5], Lynch syndrome [6], etc. Several previous studies have revealed that the PI3K/PTEN/mTOR/HIF axis [7] and the p53 tumor-suppressor system [8] might be involved in carcinogenesis. Elevated circulating levels of certain pro-inflammatory cytokines, including interleukin-6 (IL-6), interleukin-8 (IL-8), and tumor necrosis factor-α (TNF-α) have been associated with an increased risk of endometrial cancer [9, 10]. Meanwhile, a retrospective, population-based cohort study found that pelvic inflammatory disease increased the risk of developing endometrial cancer 1.79-fold [11], suggesting a potential link between chronic inflammation of the female reproductive tract and endometrial carcinogenesis.

Emerging evidence has revealed that the microbiota is increasingly considered an important factor associated with tumor development [12, 13], including endometrial cancer [14]. Several cross-sectional studies have shown alterations in the vaginal and endometrial microbiota of women with endometrial cancer [15-18]. Moreover, correlation analyses have found the microbiota to be involved in a proinflammatory response response [19] or fibrin degradation [20] in endometrial cancer, thus contributing to carcinogenesis. Another study based on endometrial organoids also demonstrated that a distinct bacterium, *L. crispatus*, may exert an anti-proliferative effect [21].

Although a continuum of microbiota throughout the female reproductive tract from the vagina to the ovaries have been reported in previous studies [22, 23], most of the research has been focused on vaginal or endometrial microbiota in endometrial cancer patients. Few recent experiments have shown the microbiota composition along the whole reproductive system in women with endometrial cancer, especially in Chinese cohorts. In this study, we systematically sampled the microbiota from five anatomical sites to investigate the microbiota continuum along the female reproductive tract. Furthermore, we explored the similarities and differences in microbial composition at each site with an aim to elucidate the role of microbiota in endometrial carcinogenesis and identify potential biomarkers for early diagnosis.

## Materials and Methods

### Participant Selection

This study was conducted at the First Affiliated Hospital, Zhejiang University School of Medicine, from December 2021 to March 2023. Women who had undergone hysterectomy by standard laparoscopic surgery for benign disease, hyperplasia, or any stage of endometrial cancer were enrolled voluntarily. Pathological diagnosis and staging were performed by an experienced gynecologist according to the FIGO 2009 guidelines [24]. The exclusion criteria were as follows: (a) pregnant women and nursing mothers; (b) history of genital tract infection or medication within 3 months; (c) individuals who used systemic antibiotics, corticosteroids, or any other immunosuppressive therapy within 3 months; and (d) individuals receiving preoperative chemotherapy or radiotherapy. All participants provided written informed consent, and they or their legally authorized caregivers were informed of the purpose of this study.

### Sample Collection

We took samples from five locations (vaginal swabs swabs, uterine swabs, fallopian tube swabs, peritoneal fluid, and endometrial tissues) to explore the microbiota diversity and similarity in different parts of female reproductive tract. All surgical procedures were performed exclusively by a single attending surgeon in a sterile operating room. All cotton swabs, surgical blades, spinal needles, and syringes used during sampling were manufactured by the same supplier in the same production lot and sterilized using standardized protocols. The vaginal swabs were collected prior to the standard pre-surgical betadine douche as follows: the vagina and the cervix were exposed to a disposable sterile vaginal speculum, and a cotton swab was gently rotated across the vaginal wall for 15 sec to absorb vaginal secretions [23]. Peritoneal fluid was aspirated from the Pouch of Douglas following injection of 10 ml sterile saline into the peritoneal cavity. All enrolled patients underwent laparoscopic hysterectomy with bilateral salpingectomy. Immediately upon excision, specimens were bisected using sterile surgical blades under aseptic conditions. Blood contamination was meticulously prevented during specimen collection. Fallopian tube samples were obtained from the ampullary region, while uterine swabs were collected from the uterine fundus [22]. After removing the corrupted and erosive tissues of the surface, the residual endometrial tissues were macrodissected. Endometrial tissues were then sectioned into fragments (approximating soybean size) with sterile blades. [20]. All specimens were immediately snap-frozen in the liquid nitrogen vapor phase before being transferred to -80°C freezers within 30 min of collection.

The remaining uterine tissue was used for pathological diagnostic and immunohistochemistry analysis conducted by an experienced gynecologist under strict aseptic procedures. The positive expression of certain receptors was scored as follows: Cells with <10% staining were scored as negative staining (-, 1); cells with 10-49% staining were scored as (+, 2); cells with 50-74% staining were scored as (++, 3); and cells with 75-100% staining were scored as (+++, 4). The staining color was scored as light-yellow particle (1), brown-yellow particle (2), and brown particle (3). The final score was defined as staining number score multiplied by staining color score [25].

### Bacterial DNA Extraction

The cotton tips of swabs were clipped with sterile surgical scissors. The peritoneal fluid was centrifuged at 3,000 ×*g* for 30 min. The swabs and the precipitates of peritoneal fluid were vortexed thoroughly to resuspend the samples in 1.5 ml of PBS (pH 7.2) and centrifuged at 12,000 ×*g* for 5 min at 25°C [23]. Then, the supernatant was removed and the resulting precipitates were used for DNA extraction using a QIAamp DNA Mini Kit (QIAGEN, Germany) following the manufacturer’s instructions with minor modification and according to previously established protocol [23, 26]. Following that, 200 mg of endometrial tissues were used for DNA extraction using a QIAamp PowerFecal DNA Kit (QIAGEN) according to manufacturer’s protocol [27]. The concentration of extracted DNA was determined by using a NanoDrop ND-1000 spectrophotometer (Thermo Electron Co., USA). All DNA was stored at -80°C before further analysis.

### 16S Library Construction

The isolated bacterial DNA was used as a template for PCR amplification of the V4 region of the bacterial 16S ribosomal RNA gene with the primer set 515F (5'-GTGCCAGCMGCCGCGGTAA-3') and 806R (5'-GGACTACHVGGGTWTCTAAT-3') [28]. Thermal cycling consisted of an initial denaturation at 98°C for 1 min, followed by 30 cycles of denaturation at 98°C for 10 sec, annealing at 50°C for 30 sec, and elongation at 72°C for 30 sec and 72°C for 5 min. The library was checked with Qubit and real- time PCR for quantification and bioanalyzer for size distribution detection. Then, the equimolar concentrations of the PCR products were pooled and sequenced using an Illumina MiSeq platform according to the manufacturer’s recommendations.

### Bioinformatic Analysis

Paired-end reads were assigned to samples based on their unique barcode which was then truncated along with the primer sequence and finally merged using Flash (v1.2.1 1) [29]. Quality filtering on the raw tags was performed using the Fastp (v0.23.1) software to obtain high-quality Clean Tags [30]. The tags were compared with the reference database (Silva) to detect chimera sequences, and then the chimera sequences were removed. For the Effective Tags obtained previously, denoising was performed with DADA2 or deblur module in QIIME2 (v2022.02) to obtain initial ASVs (Amplicon Sequence Variants) [31]. Species annotation was also performed using QIIME2. Feature tables underwent total sum scaling to generate relative abundance profiles, and rarefaction to the minimum sequencing depth across samples was performed for downstream alpha diversity analysis. Alpha diversity was calculated from 3 indices (Chao1, Simpson, and Shannon) in QIIME2. The weighted UniFrac distance matrix was also performed using QIIME2 software [32]. Finally, the LEfSe and MetaStat method was used to characterize the taxa with statistical significance and biological relevance.

### Statistical Analysis

Statistical analysis was performed by SPSS Statistics (v27.0), GraphPad Prism (v10.0), and R software (v4.0.3). Categorical variables underwent Fisher's exact test for group comparisons. Continuous variables were first assessed for normality using the Shapiro-Wilk test. Normally distributed two-group comparisons used Student's *t*-test (two-tailed), with non-normal distributions analyzed via Mann-Whitney U test. Multi-group analyses employed one-way ANOVA with Tukey post-hoc (when normality and homoscedasticity confirmed) or Kruskal-Wallis with Dunn's correction. Correlations were quantified using Spearman's rank correlation coefficient (ρ), with 95% confidence intervals. A random forest model was generated by the “random Forest” package in R software to analyze key species that distinguish the EC group from the control group [28]. The receiver operating characteristic (ROC) curves were calculated to evaluate the predictor performance of the final model.

## Results

### Clinical Characteristics of the Study Population

A total of 22 participants were included in the endometrial cancer (EC) group, 8 in the endometrial hyperplasia (Hyper) group, and 29 in the benign (Ben) group. All diagnoses were confirmed through final surgical pathology. The clinical characteristics of the cohort are presented in Table 1. There were no significant differences in BMI, age, and menstrual status between the Ben and EC groups, which suggested that the benign controls were primarily matched with the EC participants. In addition, analysis of the EC-related clinical indicators showed a significantly higher prevalence of hypertension history (*p* = 0.029) in the EC group compared to the benign group, and fasting plasma glucose (*p* = 0.021) and fibrinogen levels (*p* = 0.0003) were significantly elevated in the EC patients compared to the benign controls. There was also an increase in history of diabetes in endometrial cancer, but it was not statistically significant.

### Characterization of Microbial Communities in Different Parts of the Female Reproductive Tract

We collected samples from five distinct anatomical sites, regarding the enrolled participants, to investigate the microbiota continuum along the female reproductive tract. However, due to low read counts, some uterine swabs, fallopian tube swabs, and peritoneal fluid samples were excluded from the analysis. Therefore, a total of 58 vaginal swabs, 39 fallopian swabs, 16 peritoneal fluid samples, 36 urine swabs, and 34 endometrium samples were finally recruited.

**Fig. 1:**
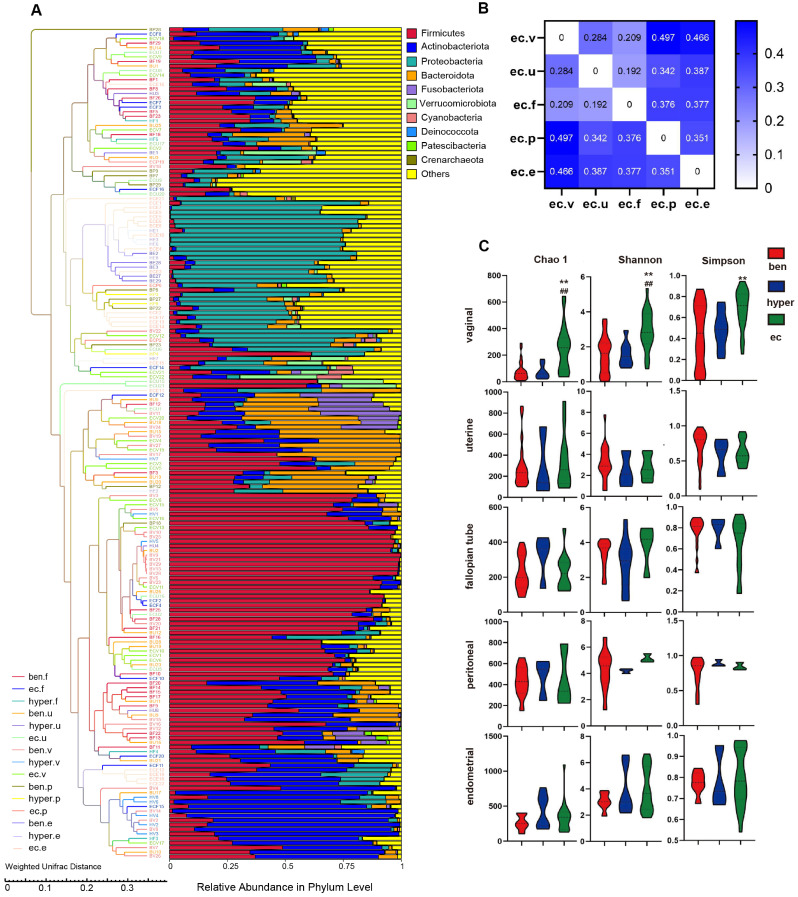
Microbial diversity in different regions of benign, hyperplasia and EC participants. (**A**) UPGMA (Unweighted Pair-group Method with Arithmetic Mean) clustering tree based on weighted UniFrac distance. Note: Left is the UPGMA clustering tree structure, and on the right is a plot of the relative abundance distribution of species at the phylum level. (**B**) Heatmap of organ correlation based on weighted UniFrac distance. (**C**) a-Diversity comparison between different disease states in the vaginal, peritoneal, fallopian tube, uterine, endometrial microbiome. Note: Ben: benign, Hyper: hyperplasia, EC: endometrial cancer. **p-value < 0.01 EC group vs. benign group; ^##^*p*-value < 0.01 EC group vs. Hyper group.

First, to investigate the impact of clinical factors on the female reproductive tract microbiota, we compared vaginal and endometrial microbial profiles across different age groups, menstrual statuses, and BMI categories among the enrolled participants. As shown in Fig. S1A-S1F, no statistically significant differences were observed in alpha-diversity (Simpson index). We then compared the Chao1, Shannon, and Simpson indices across different regions and under varying health conditions to assess microbial richness and diversity, and identify low-abundance species. As shown in Fig. 1C, the vaginal site in the endometrial cancer group exhibited a significant increase in the Chao1, Shannon and Simpson indices compared to the benign groups. In the other sites, although no statistically significant differences were observed, the alpha-diversity in the endometrial cancer group tended to be higher than that in the benign group across all three metrics. These findings suggest that the richness and diversity of the microbiota may be elevated in patients with endometrial cancer, particularly in the vaginal and endometrial regions.

To further explore the relationships among the different regions of the reproductive tract, we analyzed the weighted UniFrac distance between the five sites in endometrial cancer patients. As illustrated in Fig. 1B, the differences between the vaginal, fallopian tube, and uterine sites in the endometrial cancer group were relatively minor, while the peritoneal fluid and endometrial tissue exhibited greater divergence from the above three regions. The UPGMA clustering tree (Fig. 1A) further supported this, showing that samples were largely grouped according to their anatomical location. Notably, the endometrial tissue and peritoneal fluid shared similar microbiota structures at the phylum level, with Proteobacteria being the dominant phylum. In contrast, Firmicutes and Actinobacteria were more prevalent in the other three sites.

### Alterations in the Microecology of the Vagina and Endometrial Sites in the EC Group

**Fig. 2:**
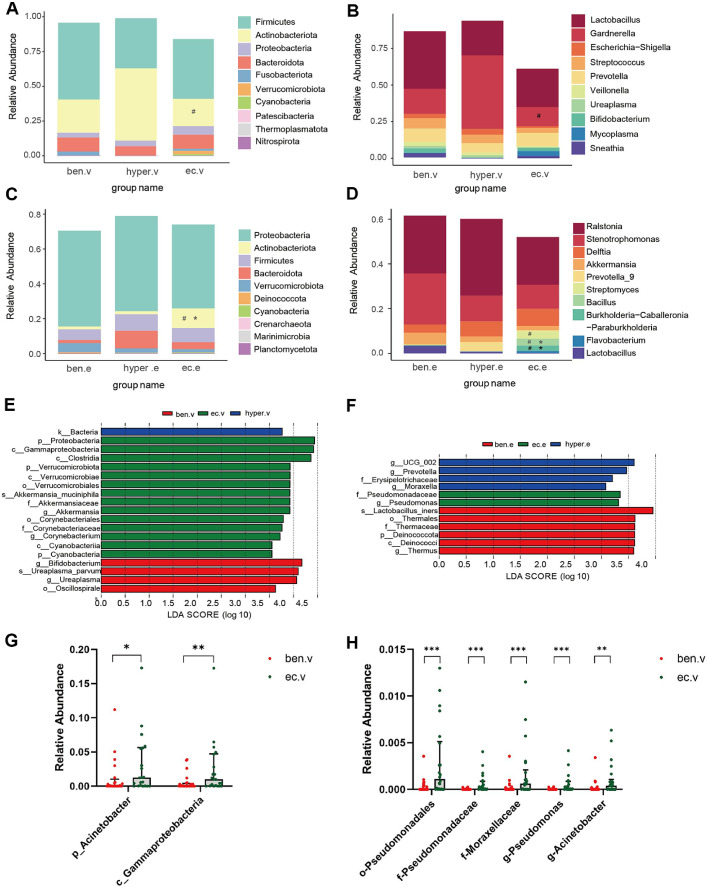
Comparison of the representative taxonomic abundance among benign, hyperplasia and EC participants. (**A**) Compositions of the bacterial community at the phylum level (top 10) between different groups in vagina. (**B**) Compositions of the bacterial community at the genus level (top 10) between different groups in vagina. (**C**) Compositions of the bacterial community at the phylum level (top 10) between different groups in endometria. (**D**) Compositions of the bacterial community at the genus level (top 10) between different groups in endometria. (**E**) LEfSe analysis showed the key discriminative biomarkers with linear discriminant analysis (LDA) score with log 10 scale > 3.5 in vaginal microbiome between groups. (**F**) LEfSe analysis showed the key discriminative biomarkers with linear discriminant analysis (LDA) score with log 10 scale > 3.0 in endometrial microbiome between groups. (**G**) Scatter plot showing the distribution of relative abundance of phylum *Acinetobacter* and class *Gammaproteobacteria* among EC group and benign group. (**H**) Scatter plot showing the distribution of relative abundance of order *Pseudomonadales* and its taxa among EC group and benign group. Note: Ben: benign; Hyper: hyperplasia; EC: endometrial; v: vaginal; e: endometrial; p: phylum; c: class; o: order; f: family; g: genus; s: species; *p-value < 0.05, **p-value < 0.01, ***p-value < 0.001, EC group vs benign group; ^#^*p*-value < 0.05 EC group vs. Hyper group.

To assess microbiota alterations in patients with hyperplasia and endometrial cancer, we conducted a taxonomic analysis at both the phylum and genus levels, focusing on the vaginal and endometrial sites. The top 10 most abundant phyla and genera for each group are presented in the cumulative bar plots (Fig. 2A-2D), and the relative abundance values for each phyla and genera are displayed in Tables S1-S4.

In the vaginal site, Firmicutes and Actinobacteriota were the most abundant phyla. Compared to the hyperplasia group, patients with EC showed a significant decrease in Actinobacteriota (19.6% vs. 51.9%, *p* = 0.04)(Fig. 2A, Table S1). As depicted in Fig. 2B and Table S2, this decrease in Actinobacteriota in EC patients was primarily due to a reduced abundance of *Gardnerella* (13.2% vs. 50.2%, *p* = 0.03), a key species associated with bacterial vaginosis [33]. Further analysis of low-abundance species revealed significant alterations in genus *Acinetobacter* and class Gammaproteobacteria between the benign and EC groups in the vaginal microbiota (Fig. 2G), which was primarily due to the enrichment of the opportunistic pathogen *Pseudomonadales* in EC group, along with changes at the family and genus levels (Fig. 2H). Specifically, *Acinetobacter* (0.11% vs. 0.02%, *p* = 0.006) and *Pseudomonas* (0.05% vs. 0.0003%, *p* < 0.001) were significantly enriched in EC patients compared to the benign group. LEfSe analysis identified the phylum Verrucomicrobiota, including the class Verrucomicrobiae, order Verrucomicrobiales, family *Akkermansiaceae*, genus *Akkermansia*, and species *Akkermansia muciniphila*, as significantly enriched in the EC group at the vaginal site (Fig. 2E).

At the endometrial site, Proteobacteria was the most abundant phylum. Notably, the phylum Actinomyces was significantly more enriched in the EC group compared to the other two groups (EC vs. Ben: 11.2% vs. 1.6%, *p* = 0.03; EC vs. Hyper: 11.2% vs. 1.7%, *p* = 0.03). Additionally, Cyanobacteria was more prevalent in the EC group than in the benign group (0.33% vs. 0.05%, *p* = 0.04) (Fig. 2C, Table S3). At the genus level, *Bacillus* was significantly more abundant in the EC group compared to both the benign and hyperplasia groups (EC vs. Ben: 3.15% vs. 0.21%, *p* = 0.04; EC vs. Hyper: 3.15% vs. 0.04%, *p* = 0.046), as was *Burkholderia-Caballeronia-Paraburkholderia* (EC vs. Ben: 2.45% vs. 0.02%, *p* = 0.04; EC vs. Hyper: 2.45% vs. 0.00%, *p* = 0.04). Additionally, *Streptomyces* was significantly enriched in the EC group compared to the hyperplasia group (3.45% vs. 0.02%, *p* = 0.04) (Fig. 2D, Table S4). Interestingly, the family *Pseudomonadaceae* and genus *Pseudomonas*, which were enriched in the vaginal microbiota of EC patients, were also identified as distinct taxa in the endometrium when compared to the other two groups by LEfSe analysis (Fig. 2F).

### Microecological Changes and Related Clinical Indicators

**Fig. 3:**
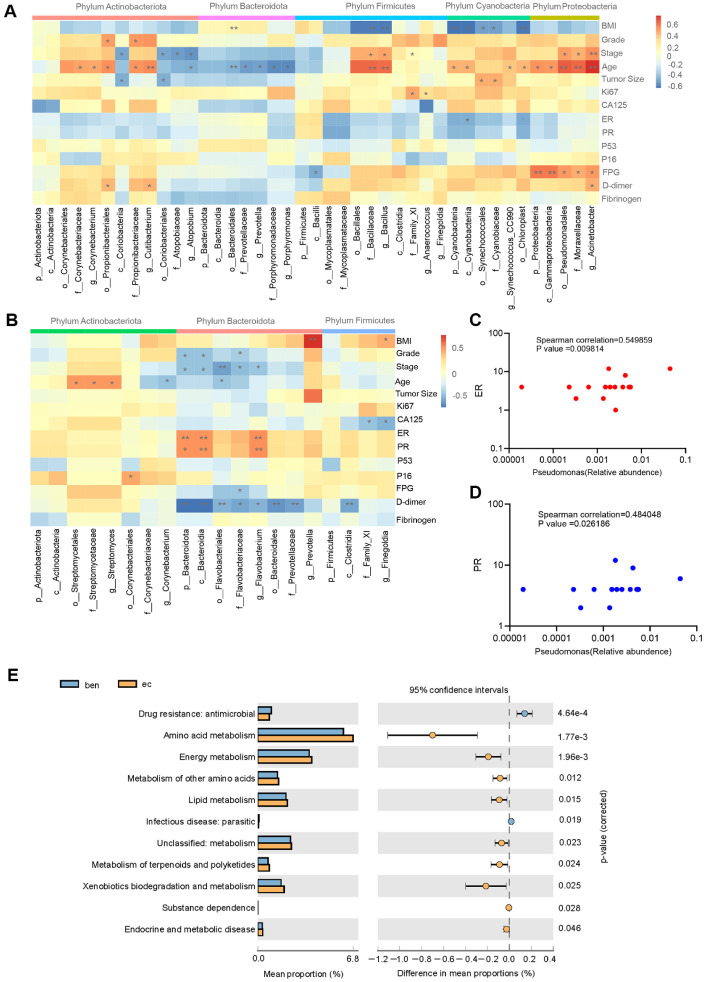
Associations between microbiota and clinical indicators in patients with EC. (**A**) Heatmap of correlation analysis between the vaginal microbiota and clinical indicators. (**B**) Heatmap of correlation analysis between the endometrial microbiota and clinical indicators. (**C-D**) Scatter plots of the relative abundance of *Pseudomonas* in endometrial tissue and the scores of estrogen receptor positive expression in tumor tissue by immunohistochemistry. (**E**) Predicted KEGG functional pathways differences at level 2 in endometrial tissue inferred from 16S rRNA gene sequences using PICRUSt2. Note: Ben: benign; Hyper: hyperplasia; EC: endometrial; v: vaginal; BMI: body mass index; Grade: tumor differentiation grade; Stage: FIGO stage 2009; ER: estrogen receptor; PR: progesterone receptor; FPG: fasting plasma glucose.

To explore the potential role of EC-specific microbiota, we conducted a Spearman’s correlation analysis between endometrial cancer-related clinical characteristics and the microbiota in the vagina (Fig. 3A) and endometrium (Fig. 3B) of the EC group. The results indicated that a higher relative abundance of the endometrial genus *Flavobacterium* and its related taxa were significantly correlated with earlier FIGO stage and lower serum D-dimer levels. In the vaginal microbiota, a higher relative abundance of the genus *Acinetobacter*, which was significantly enriched in EC patients, was associated with elevated fasting plasma glucose (FPG), higher serum D-dimer levels, advanced age, and later FIGO stage. Additionally, *Pseudomonas*, a genus specifically elevated in both the vaginal and endometrial microbiota of EC patients, was positively associated in endometrium with estrogen receptor (ER) and progesterone receptor (PR) expression (Fig. 3C and 3D).

To further elucidate the mechanistic connection between microbial alterations and endometrial carcinogenesis, functional pathway prediction was performed using PICRUSt2 in endometrial microbiota, revealing differentially enriched KEGG pathways at level 2 when comparing the EC group with benign group. As shown in Fig. 3E, a total of 11 differentially abundant functional orthologs were identified. The enriched orthologs in EC patients included amino acid metabolism, energy and lipid metabolism, metabolism of terpenoids and polyketides, xenobiotics biodegradation and metabolism, substance dependence, and endocrine and metabolic disease. As demonstrated, we can see that the orthologs enriched in EC group were majorly associated with metabolic dysregulation, which is consistent with our Spearman correlation analysis. Additionally, the benign group showed significantly enriched orthologs related to drug resistance and parasitic infection.

### Random Forest Model Based on Vaginal Microbiomes to Distinguish EC Patients from Benign Controls

**Fig. 4:**
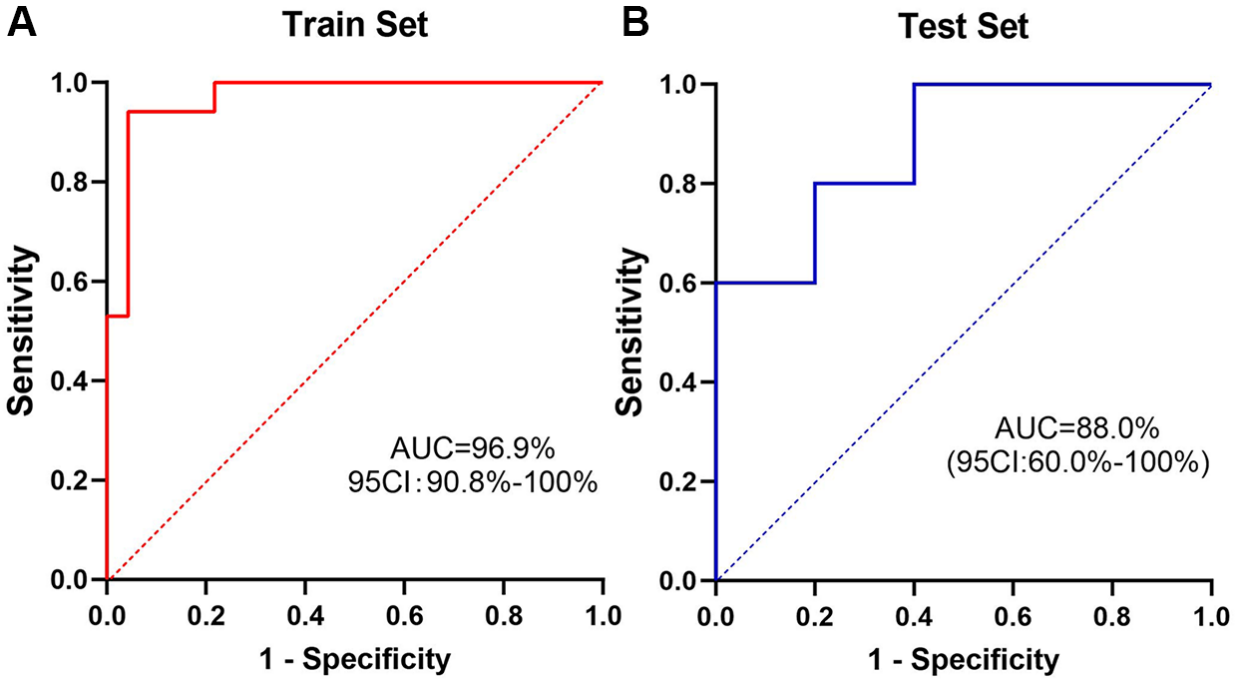
Vaginal microbiota-based classification of endometrial cancer patients and patients with benign disease. (**A**) The ROC curve for the random forest model in the train set. (**B**) The ROC curve for the random forest model in the test set.

To develop an effective method for differentiating between the EC and benign groups, and given the high accessibility of vaginal microbiota samples, we constructed a random forest model based on vaginal microbial profiles. The dataset was randomly split, with 80% used for training and 20% for testing. To establish a more effective model, the optimal "ntree" value was determined by minimizing classification error, with error rates reaching their lowest point when ntree approached 200 (Fig. S2A). Simultaneously, 10-fold cross-validation demonstrated that the model achieved peak accuracy when the feature number was set to 10 (Fig. S2B). To identify the most important microbial taxa, we calculated the mean decrease in Gini index [34], and the top markers identified at the genus level were *Mobiluncus*, *Peptoniphilus* and *Porphyromonas* (Fig. S2C). Based on these, we established the final predictive model, which demonstrated robust performance on both the training and test sets.(Fig. 4A: ROC curve of train set, AUC = 96.9%, 95% confidence interval [CI]: 90.8%-100%; Fig. 4B: ROC curve of test set, AUC = 88.0%, 95% CI: 60.0%–100%).

## Discussion

Our study demonstrated the alterations of bacterial communities throughout the female reproductive tract between endometrial cancer, endometrial hyperplasia and benign patients, and biomarkers were found to distinguish endometrial cancer patients from benign patients. Meanwhile, the genera specifically enriched in EC patients were positively associated with a high level of fasting plasma glucose and the expression of estrogen receptor in endometrium tissue. The novel results of this study offer a better understanding of this disease and provide new markers for diagnosis.

In the past few years, a growing number of studies have indicated the existence of a distinct bacterial community continuum along the female reproductive tract [22], and the alterations of bacterial colonization in the genital tract have been reported in endometrial cancer patients [15, 16, 19]. In this study, we examined the bacterial diversity across five sites: the vagina, uterus, fallopian tube, peritoneal, and endothelial tissues. To our knowledge, ours is one of the first studies to characterize the pelvis microbiota in endometrial cancer patients. Interestingly, we found that endometrial tissue shares similar bacterial structures with peritoneal fluid rather than uterus fluid. It was widely accepted that the uterine microbiome stems from the vaginal microbiome since the continuum changes along the female reproductive tract, which was consistent with our findings that the bacterial structures of the vaginal, fallopian tube and uterine microbiome were relatively similar. Meanwhile, Yang and colleagues observed a higher EC risk in the pelvic inflammatory disease cohort [11], which indicated that the pelvic environment could affect the endometrial microbiota. However, in our study, some peritoneal fluid samples were excluded from analysis due to low read counts, and therefore further exploration is necessary. We found that in EC patients, the genera *Acinetobacter* and *Pseudomonas* were significantly enriched in the vagina, and *Pseudomonas* was also enriched in endothelial tissues. Previous studies have reported *Acinetobacter* and *Pseudomonas* as the dominant microbes in the endometrial samples of 25 women who had undergone a total hysterectomy for fibroids or endometrial hyperplasia [35]. LEfSe analysis identified *Akkermansia muciniphila* in the vagina of EC patients. This bacterium has garnered attention for its potential role in reducing abnormal inflammatory responses and metabolic disorders [36], including obesity [37], diabetes [38, 39], inflammatory bowel disease [28], psychiatric diseases [40], aging [41], and other diseases. As an essential component of gut microbiota, *Akkermansia* was previously reported to be reduced in the vaginal microbiota of bacterial vaginosis patients [42]; however, we are the first to observe its enrichment in vaginal samples from EC patients. Meanwhile, we also found that *Mobiluncus*, *Peptoniphilus*, and *Porphyromonas* in the vagina were critical microbial markers for EC. *Peptoniphilus* and *Porphyromonas* were members of the so-called Anaerobic Bacteria Biomarkers Set (ABBS) [43], which is associated with a variety of cancers, such as high-grade prostate cancer [44], bladder cancer [45], breast cancer [46], and even endometrial cancer. Previous studies have found high abundance of *Peptoniphilus* in endometrial cancer tissues [18], and *Peptoniphilus* was also enriched in vaginal microbiota of endometrial and cervical cancer patients compared to controls with no cancer [47]. Moreover, *Porphyromonas* has been reported to be an indicator of EC [15, 16], which is consistent with our study.

Microecological disorders have been demonstrated to play a role in several aspects of endometrial carcinogenesis, above chronic inflammation [19, 21], fibrin degradation [20], and microenvironment [15]. In our research, we unexpectedly found that microecological disorders might be involved in metabolic disorders, including those involving estrogen and glucose. Insulin resistance and hyperinsulinemia, features of type 2 diabetes, increase the bioavailability of estrogen and insulin-like growth factor (IGF)-1, which promote endometrial proliferation [48] and are conducive to the development of endometrial cancer (especially type I) [49]. In addition, we also found that *Pseudomonas*, which might be stimulated by elevated levels of estrogen, was enriched in the vagina of ER and PR positive EC patients [50]. Uduwela and colleagues have revealed that arylsulfatase from *Pseudomonas* was able to enhance the activity of steroid sulfatase [51], which is capable of catalyzing sulfated steroid precursors to the free steroid, and *Pseudomonas* has been reported in other estrogen-related diseases, such as endometriosis [52], which is also consistent with our findings. Furthermore, *Acinetobacter*, distinctly enriched in vagina in EC group in our study, was highly associated with hyperglycemia. Past research has shown that *Acinetobacter* increased in ocular surface microbiota in type 2 diabetes mellitus patients [53], as well as in the gut microbiota in metabolic syndrome rats [54], which was considered a symptom of chronic inflammation.

In summary, our study identified specific alterations in the composition and diversity of the reproductive tract microbiota in endometrial cancer patients. The findings suggest that microbiota dysbiosis may be involved in the metabolic dysregulation of endometrial cancer. Moreover, using a diagnostic model based on specific bacterial biomarkers, we demonstrated strong predictive value, which offers potential implications for the early diagnosis and mechanistic understanding of endometrial cancer.

Our study has some limitations. First, there was no healthy cohort among our research participants, and our sample size was relatively small. In addition, our samples were taken from patients via laparoscopy, which could potentially contaminate the samples [21]. Second, while there is a possibility that the alterations of microbiota may play a role in metabolic disorder limitation, further longitudinal study is necessary to explore how this works. Despite these limitations, our conceptually novel study provides important insight into EC.

## Supplemental Materials

Supplementary data for this paper are available on-line only at http://jmb.or.kr.



## Figures and Tables

**Table 1:** Demographic and clinical parameters of subjects of the study cohorts.