# Supplementary material for: Distinct Microbial Signatures along the Female Reproductive Tract in Endometrial Cancer Patients
Source: J Microbiol Biotechnol. 2025 Aug 26;35:e2503048. doi: 10.4014/jmb.2503.03048 (PMC12409432; doi:10.4014/jmb.2503.03048)
Supplement: Supplementary file 1 [file jmb-35-e2503048-supple.pdf]

J. Microbiol. Biotechnol. 2025. 35: e2503048
https://doi.org/10.4014/jmb.2503.03048

Supplementary Figures and Tables

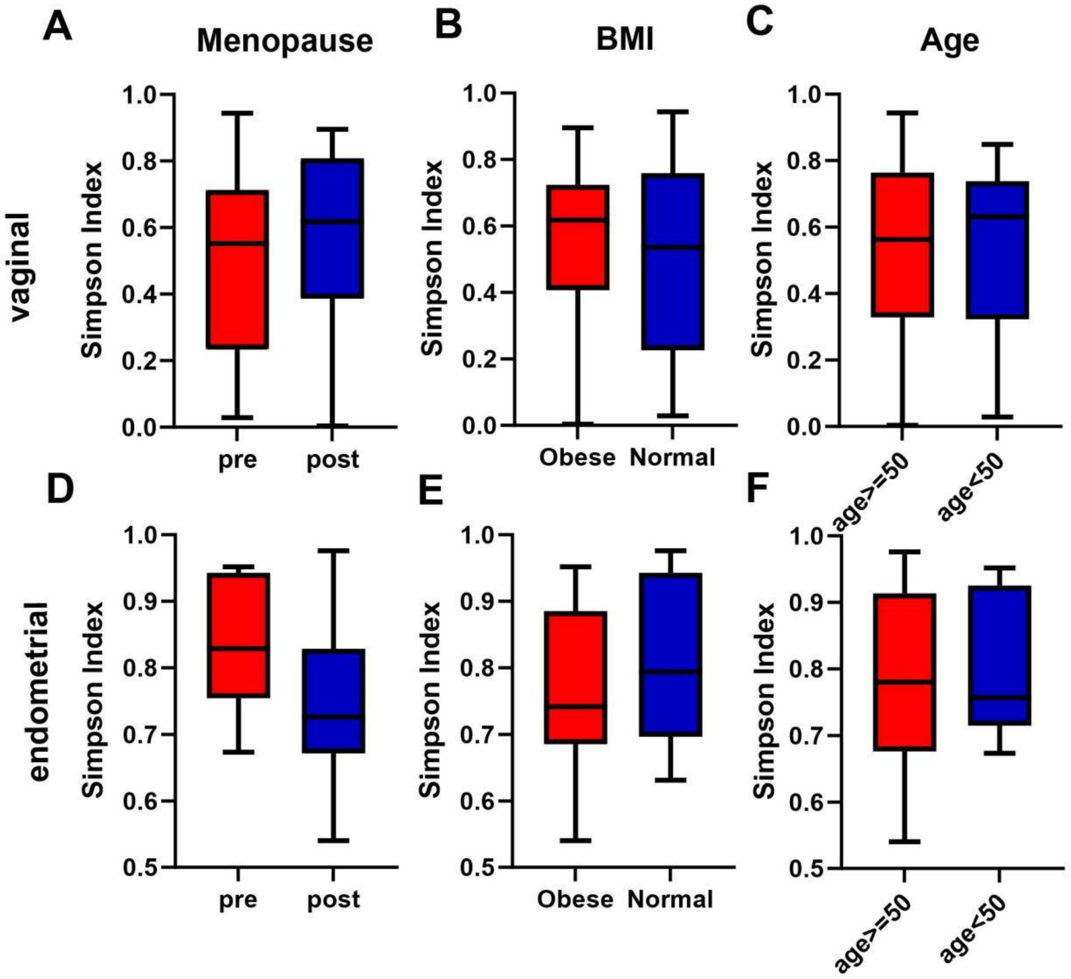

Fig. S1. Influence of clinical factors on bacterial community diversity among individuals. **(A)** a-Diversity (Simpson index) comparison between premenopausal and postmenopausal woman in vaginal microbiome. **(B)** a-Diversity (Simpson index) comparison between obese (BMI>=24 kg/m$^{2)}$ and normal BMI (18.5 kg/m^2 < BMI < 24 kg/m^2) individuals in vaginal microbiome. (**C**) a-Diversity (Simpson index) comparison between age>=50years and age<50 years individuals in vaginal microbiome. (**D**) a-Diversity (Simpson index) comparison between premenopausal and postmenopausal woman in endometrial microbiome. **(E)** a-Diversity (Simpson index) comparison between obese (BMI>=24 kg/m2) and normal BMI (18.5 kg/m2 < BMI < 24 kg/m2) individuals in endometrial microbiome. **(F)** a-Diversity (Simpson index) comparison between age>=50years and age<50 years individuals in endometrial microbiome.

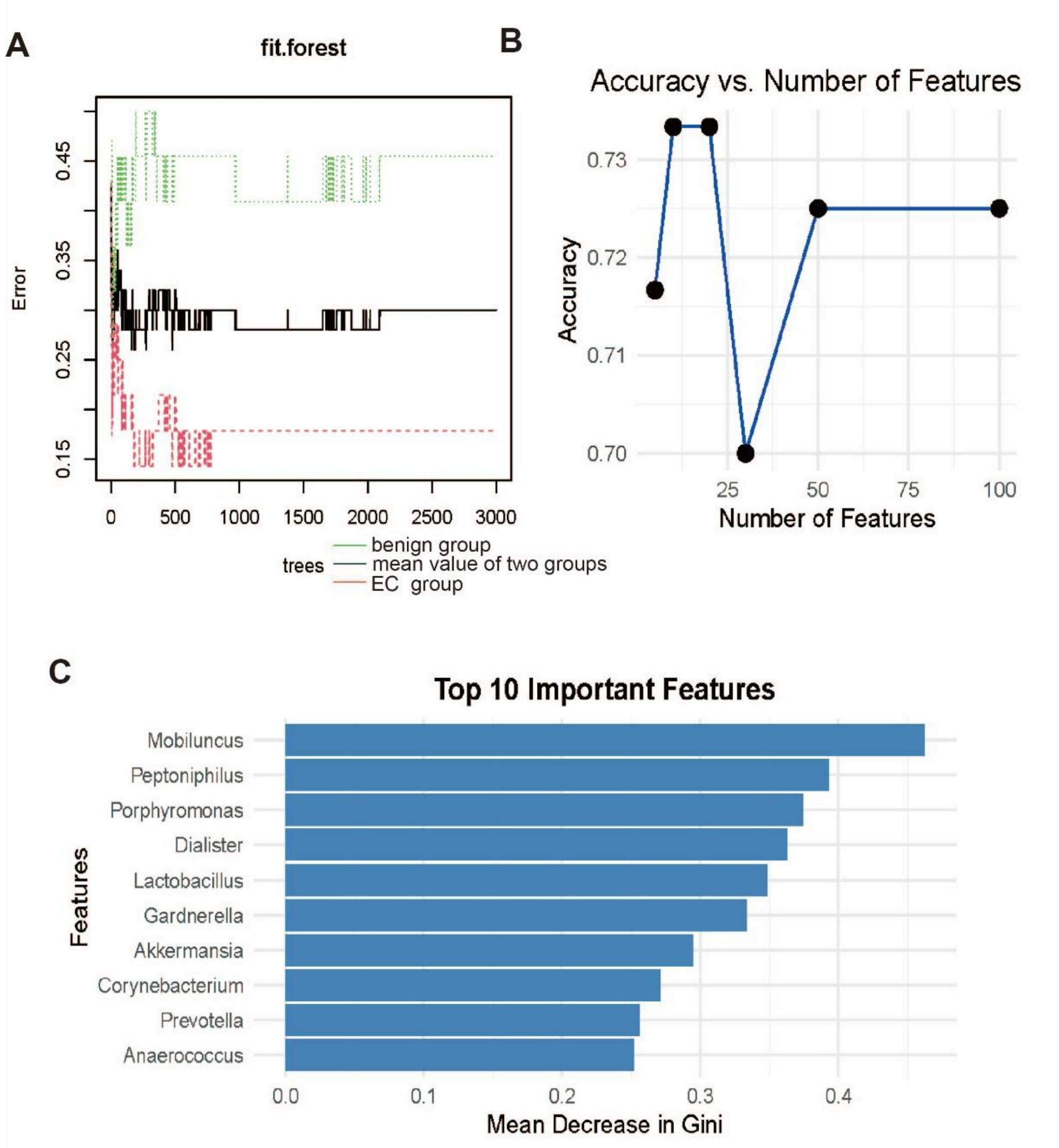

Fig. S2. (A) The quantitative relationship between "ntree" and classification error rate. (B) Accuracy of the training set under different numbers of feature variables obtained via 10-fold cross-validation. **(C)** The top 10 genera in Mean Decrease Gini.

Table S2. Relative abundance of the bacterial community (mean ± SD) at the phylum level (top 8) between different groups in vaginal microbiome. # p-value < 0.05 EC group vs hyper group.

	ben.v	hyper.v	ec.v
Firmicutes	0.557±0.355	0.360±0.347	0.430±0.305
Actinobacteriota	0.238±0.277	0.519±0.374	0.196±0.159#
Proteobacteria	0.0350±0.154	0.0406±0.0739	0.0613±0.156
Bacteroidota	0.101±0.177	0.0686±0.115	0.101±0.148
Fusobacteriota	0.0286±0.0842	0.000422±0.00119	0.0156±0.0409
Verrucomicrobiota	0.0000275±0.000130	0.0000172±0.0000485	0.0235±0.0736
Cyanobacteria	0.000380±0.00156	0.0000995±0.000148	0.0102±0.0312
Patescibacteria	0.000878±0.00314	0.0000206±0.0000481	0.00104±0.00223

Table S2. Relative abundance of the bacterial community (mean ± SD) at the genus level (top 10) between different groups in vaginal microbiome. # p-value < 0.05 EC group vs hyper group.

	ben.v	hyper.v	ec.v
Lactobacillus	0.393±0.441	0.237±0.370	0.262±0.340
Gardnerella	0.177±0.267	0.502±0.386	0.132±0.164#
Escherichia-Shigella	0.0298±0.154	0.0384±0.0745	0.00895±0.0366
Streptococcus	0.0648±0.164	0.0588±0.159	0.0348±0.0704
Prevotella	0.0981±0.174	0.0588±0.105	0.0899±0.146
Veillonella	0.0234±0.0908	0.0176±0.0432	0.00997±0.0285
Ureaplasma	0.0220±0.0890	0.0168±0.0275	0.000523±0.00121
Bifidobacterium	0.0311±0.114	0.00182±0.00256	0.0248±0.0794
Mycoplasma	0.00295±0.0151	0.000292±0.000825	0.0330±0.108
Sneathia	0.0284±0.0843	0.000378±0.00107	0.0154±0.0410

Table S3. Relative abundance of the bacterial community (mean ± SD) at the phylum level (top 8) between different groups in endometrial microbiome. # p-value < 0.05 EC group vs hyper group.* p-value < 0.05 EC group vs ben group.

	ben.e	hyper.e	ec.e
Proteobacteria	0.550±0.247	0.547±0.267	0.482±0.240
Actinobacteriota	0.0159±0.0164	0.0174±0.0231	0.112±0.180*#
Firmicutes	0.0621±0.0743	0.0946±0.119	0.0820±0.103
Bacteroidota	0.0165±0.00924	0.101±0.142	0.0392±0.0755
Verrucomicrobiota	0.0528±0.127	0.0255±0.0607	0.0199±0.0446
Deinococcota	0.00684±0.0164	0.0000579±0.0000896	0.0000257±0.0000931
Cyanobacteria	0.000479±0.00101	0.00262±0.00475	0.00330±0.00568*
Crenarchaeota	0.00017±0.000279	0.000161±0.000394	0.00094±0.000863

Table S4. Relative abundance of the bacterial community (mean ± SD) at the genus level (top 10) between different groups in endometrial microbiome. # p-value < 0.05 EC group vs hyper group.* p-value < 0.05 EC group vs ben group.

	ben.e	hyper.e	ec.e
Ralstonia	0.258±0.147	0.344±0.203	0.213±0.234
Stenotrophomonas	0.229±0.123	0.114±0.109	0.108±0.131
Delftia	0.0368±0.0242	0.0676±0.0370	0.0768±0.0847
Akkermansia	0.0528±0.127	0.0252±0.0605	0.0191±0.0438
Prevotella_9	0.000466±0.000421	0.0413±0.0966	0.00234±0.00522
Streptomyces	0.00233±0.00525	0.000177±0.000172	0.0345±0.0703#
Bacillus	0.00207±0.00428	0.000427±0.000605	0.0315±0.0631*#
Burkholderia-Caballeronia-Paraburkholderia	0.000241±0.000590	0.00000963±0.0000236	0.0245±0.0504*#
Flavobacterium	0.000373±0.000886	0.00227±0.00521	0.00879±0.0369
Lactobacillus	0.0334±0.0639	0.00624±0.00986	0.00140±0.00412
